# Does more sequence data improve estimates of galliform phylogeny? Analyses of a rapid radiation using a complete data matrix

**DOI:** 10.7717/peerj.361

**Published:** 2014-04-22

**Authors:** Rebecca T. Kimball, Edward L. Braun

**Affiliations:** Department of Biology, University of Florida, Gainesville, FL, USA

**Keywords:** Galliformes, Rapid radiation, Sampling strategies, Data matrix size

## Abstract

The resolution of rapid evolutionary radiations or “bushes” in the tree of life has been one of the most difficult and interesting problems in phylogenetics. The avian order Galliformes appears to have undergone several rapid radiations that have limited the resolution of prior studies and obscured the position of taxa important both agriculturally and as model systems (chicken, turkey, Japanese quail). Here we present analyses of a multi-locus data matrix comprising over 15,000 sites, primarily from nuclear introns but also including three mitochondrial regions, from 46 galliform taxa with all gene regions sampled for all taxa. The increased sampling of unlinked nuclear genes provided strong bootstrap support for all but a small number of relationships. Coalescent-based methods to combine individual gene trees and analyses of datasets that are independent of published data indicated that this well-supported topology is likely to reflect the galliform species tree. The inclusion or exclusion of mitochondrial data had a limited impact upon analyses upon analyses using either concatenated data or multispecies coalescent methods. Some of the key phylogenetic findings include support for a second major clade within the core phasianids that includes the chicken and Japanese quail and clarification of the phylogenetic relationships of turkey. Jackknifed datasets suggested that there is an advantage to sampling many independent regions across the genome rather than obtaining long sequences for a small number of loci, possibly reflecting the differences among gene trees that differ due to incomplete lineage sorting. Despite the novel insights we obtained using this increased sampling of gene regions, some nodes remain unresolved, likely due to periods of rapid diversification. Resolving these remaining groups will likely require sequencing a very large number of gene regions, but our analyses now appear to support a robust backbone for this order.

## Introduction

Continuing improvements in data collection and methods of phylogenetic analyses have revolutionized our understanding of evolutionary relationships (Delsuc, Brinkmann & Philippe, 2005). However, many relationships in the tree of life remain in question despite intensive study. These difficult to resolve relationships, sometimes called “bushes” in the tree of life (Rokas & Carroll, 2006), are thought to reflect rapid evolutionary radiations. Some of these cases may reflect true hard polytomies (simultaneous speciation events), though others may represent soft polytomies that could be resolved if datasets of sufficient size are collected and analyzed appropriately. Questions remain about the best methods to resolve these difficult problems (e.g., Patel, Kimball & Braun, 2013). While taxon sampling may improve phylogenetic resolution in some cases, in others more data per species has to potential to yield greater improvements to resolution than adding in data from additional species (reviewed by Nabhan & Sarkar, 2012).

The avian order Galliformes, informally called gamebirds or landfowl, includes some of the best-studied avian species. This includes the economically important chicken and turkey, the first two avian genomes sequenced (Dalloul et al., 2010; Hillier et al., 2004), as well as other important model systems such as the Japanese quail (e.g., Poynter, Huss & Lansford, 2009). As such, there exists a large body of literature about galliform physiology, reproduction, genetics, development and behavior. This order, which likely arose in the Cretaceous (Brown et al., 2008; Pereira & Baker, 2006), has also been extensively studied phylogenetically, and recent studies have typically separated the order into five families (reviewed in Wang et al., 2013). The largest family, the Phasianidae, which underwent its initial radiation between 40 to 65 million years ago (Brown et al., 2008; Pereira & Baker, 2006), still has many relationships that have remained problematic. These problematic relationships include the placement of some of the key avian model systems (i.e., the chicken, turkey, and Japanese quail). Indeed, the placement of many key phasianid taxa has varied among recent studies. This is likely to reflect the existence of a number of very short internodes within Phasianidae (e.g., Kimball, St Mary & Braun, 2011), suggesting that rapid radiations may have occurred and that these radiations are likely to have led to some of the difficulties in resolving relationships within this group. Analysis of supermatrices, constructed either by combining sequence data from multiple gene regions (Kimball, St Mary & Braun, 2011) or by combining sequence data with morphological and behavioral traits (Crowe et al., 2006), also exhibited limited support for a number of relationships. Those studies included more taxa than previous studies but they used data matrices with substantial amounts of missing data. To explore the utility of taxon sampling with a more consistent sampling of genes, Wang et al. (2013) sequenced six nuclear loci and two mitochondrial regions for 88 galliforms. This improved the support for some relationships and clarified the position of several previously poorly sampled lineages, but it provided limited improvement to the backbone phylogeny of the Phasianidae. These remaining unresolved and poorly supported nodes make it difficult to place the large body of literature about galliforms into an evolutionary framework.

Simulations based on parameters estimated from avian data demonstrate that increasing partition size leads to more accurate resolution of short internodes and that analyses of introns yields better results (with smaller amounts of sequence data) than that of exons (Chojnowski, Kimball & Braun, 2008). Those simulations were focused on the power of a single locus to resolve gene trees rather than the power of a multi-locus dataset to resolve a species tree. However, it seems reasonable to postulate that sequencing additional unlinked loci will improve phylogenetic resolution. It is also likely to prove beneficial to include mitochondrial sequence data in such a data matrix. Although phylogenetic analyses using avian mitochondrial sequences have proven to be sensitive to model selection and taxon sampling when applied to deep branches in the avian tree (Braun & Kimball, 2002; Pratt et al., 2009), the smaller population size for the mitochondrial genome increases the probability that the mitochondrial gene tree will match the species tree (Moore, 1995). Indeed, including both multiple unlinked nuclear loci along with mitochondrial sequences has been shown to improve phylogenetic estimation under some circumstances (Corl & Ellegren, 2013; Sanchez-Gracia & Castresana, 2012). On the other hand, Kimball et al. (2013) found that increasing the number of sites in a supermatrix nearly two-fold to examine higher-level avian relationships did not improve bootstrap support or resolve additional nodes, suggesting there may be limits to the improvement that can be obtained with larger datasets (although truly genome-scale datasets have been analyzed for only a few clades in the tree of life). This raises the question of whether additional data could substantially improve resolution for the rapid radiations in the galliforms.

Here we collected data from 15 nuclear loci and three mitochondrial regions to generate a data matrix comprising more than 15,000 sites and examined whether sampling these additional loci provided improved resolution and support within the galliforms. Our taxon sampling was focused on the Phaisanidae and specifically targeted to include several difficult to resolve parts of the galliform tree (i.e., relationships defined by short internodes). However, we also included representatives of other galliform families to provide outgroups. The data matrix was fully sampled, such that each species was represented by sequence data for each nuclear intron and mitochondrial region. Thus, missing data could not have an impact upon our conclusions. First, we used this dataset to determine whether we could improve our understanding of relationships among difficult nodes within the Phasianidae both by conducting analyses of concatenated sequences and using methods that incorporate the multispecies coalescent. Second, we also analyzed a subset of these taxa to allow direct comparisons to an earlier study (Kimball & Braun, 2008) that used fewer loci to directly assess the impact of adding more sequence data per species on bootstrap support. Finally, we generated jackknifed datasets of varying size from the concatenated data to further explore the impact of increasing dataset size on phylogenetic reconstruction in galliforms.

## Methods

### Molecular methods

The species included in this analysis represent a range of galliform lineages, with an emphasis on the main phasianid lineages where there has been conflict among recent studies (Table S1). We combined published sequences from our earlier studies (Armstrong, Braun & Kimball, 2001; Cox, Kimball & Braun, 2007; Kimball & Braun, 2008; Kimball et al., 1999; Kimball et al., 2001; Randi et al., 2000; Randi et al., 2001; Wang et al., 2013) with novel sequences collected as part of this study. This study focuses on the same samples included in Kimball & Braun (2008), since that study included broad sampling of most of the Phasianidae, including clades that appear likely to have undergone a rapid radiation, as well as a set of taxa from the other galliform families. However, Kimball & Braun (2008) lacked a representative of one key phasianid clade, the Arborophilinae. It also did not include the argus pheasant, which was placed in an unexpected position in the Kimball, St Mary & Braun (2011) supermatrix analysis. To address these issues, this study included the crested partridge (*Rollulus rouloul*), a representative of the Arborophilinae, and the argus pheasant (*Argusianus argus*) as well as the samples from Kimball & Braun (2008).

Primers for PCR amplification and sequencing conditions are described in Cox, Kimball & Braun (2007), Kimball et al. (2009), Wang et al. (2013), and in Table S2. PCR products were amplified using standard protocols, and amplified products were cleaned for sequencing by precipitation using an equal volume of PEG:NaCl (20%:2.5M). Sequencing of PCR products was done using either ABI BigDye^®^ Terminator v.1.0, BigDye^®^ Terminator v.3.1, or Beckman DTCS Quickstart^®^ chemistries. Manufacturers’ recommendations were followed, except reaction volumes were cut to 1/2–1/6 of the recommended volume. Sequences were analyzed on an ABI Prism™ 3100-Avant genetic analyzer (PE Applied Biosystems) or a CEQ™ 8000 (Beckman-Coulter™) genetic analysis system. Double-stranded contigs were assembled using Sequencher™ 4.1 (Gene Codes Corp.).

A number of the nuclear loci were heterozygous in some species, and in some cases the alleles differed in size within an individual. These length polymorphisms made it impossible to sequence the PCR products cleanly in both directions. For this small proportion of samples, PCR products were cloned using the pGEM^®^-T Easy vector (Promega Corp.). Plasmid preparations were done using Eppendorf Perfectprep^®^ Plasmid Mini kit, and the resulting plasmids were sequenced in each direction. In these cases, the clean portions of the original sequences from the PCR products as well as those from the plasmids were used to assemble the final contig. The majority of samples did not require cloning and were sequenced directly. When we observed heterozygosities they were coded using the standard IUPAC ambiguity codes (e.g., R was used for sites that were heterozygous for A and G) to limit any biases that might be associated with choosing one allele over the other. Differences between alleles (based on the observed number of heterozygous sites, when they were present) were smaller than the differences between species.

### Alignment and phylogenetic analysis

Sequences of the mitochondrial coding regions were equal in length and did not have any insertions or deletions, so alignment was straightforward. Preliminary alignments for the nuclear introns and the 12S rRNA region were generated using ClustalX (Thompson et al., 1997) and then optimized by eye. Regions that were difficult to align with confidence were identified and excluded from analyses. Six microinversions (cf. Braun et al., 2011) in the nuclear introns were identified by eye during the alignment process. These were treated as described in Hackett et al. (2008) and excluded from analysis. A large autapomorphic insertion (an ERV transposable element insertion) in FGB intron 7 was also excluded from analyses; two synapomorphic transposable element insertions were included in analyses. Sequences collected for this study have been deposited in GenBank (KC749476–KC749685, KC749760–KC749857, KC749907–KC749953).

We examined for base composition heterogeneity using several metrics (Table S2). First, the *χ*^2^ test of deviation from base composition homogeneity, as implemented in PAUP^∗^ 4.0b10 (Swofford, 2003), was used to identify any data partitions that showed significant deviation from base composition stationarity. Second, the base composition for variable sites in each locus, parsed from the in PAUP^∗^ output for the *χ*^2^ test, were used to calculate the relative composition variability (RCV; Phillips & Penny, 2003) as a summary statistic. Finally, following Harshman et al. (2008) and Regier et al. (2013), we inspected neighbor-joining (NJ) trees based upon “base composition distances” among taxa (Euclidean distances between the vectors describing base composition) to determine whether any clusters corresponded to clades in the trees.

We used PAUP^∗^ to estimate the maximum likelihood (ML) tree on the concatenated dataset, a concatenated nuclear dataset, a concatenated mitochondrial dataset, and each individual locus or mitochondrial region. The appropriate models for ML analyses of the various concatenated matrices and for each independent nuclear locus or mitochondrial region were determined using the Akiake information criterion (AIC) using Modeltest 3.6 (Posada & Crandall, 1998). ML trees were identified using a heuristic search with 10 random sequence additions and the parameters recommended by Modeltest.

Bootstrap support using maximum likelihood was performed for the three concatenated datasets described above, as well as the individual loci or mitochondrial regions, using the GTRGAMMA model in RAxML 7.2.8 (Stamatakis, 2006) with 500 standard bootstrap replicates. Partitioned ML analyses (using each locus or mitochondrial region as a separate partition) of the multi-locus datasets were also conducted using RAxML using 500 bootstrap replicates.

To allow a direct comparison between analyses of this dataset the results of Kimball & Braun (2008), where only four nuclear loci and two mitochondrial regions were examined, we excluded two species (*Argusianus argus* and *Rollulus rouloul*) to obtain a dataset with the same species as included in that study. We also conducted additional analyses using only the data that were not used in Kimball & Braun (2008), which corresponded to 11 additional loci and a single mitochondrial region. This allowed us to obtain an independent assessment of galliform relationships. In both cases, we then obtained an ML tree using PAUP^∗^ (Swofford, 2003) and a bootstrap consensus tree, using unpartitioned and partitioned analyses, in RAxML (Stamatakis, 2006) as described above for the concatenated data for all loci and mitochondrial regions.

Bayesian analyses of the concatenated dataset were conducted using MrBayes 3.1.2 (Ronquist & Huelsenbeck, 2003). The appropriate model for each partition (locus or mitochondrial region) was determined using the AIC (limiting the set of models under consideration to those implemented in MrBayes). We used two simultaneous searches with four chains each (three heated chains and one cold chain) that were run for, 20 million generations, sampling every 1000 generations and discarding the first 2,000,000 generations as “burn-in”, which appeared sufficient in an inspection of the data. The runs appeared to converge, as the harmonic means of the different runs were similar, the posterior scale reduction factors were essentially 1, and the average deviations of the split frequencies were substantially smaller than 0.01.

We estimated a species tree from individual gene trees using two approaches. First, we used BUCKy 1.4.0 (Larget et al., 2010) to estimate both the primary concordance tree and the population (species) tree. The primary concordance tree represents a summary of those clades with the highest concordance factors, which are estimates of the proportion of sampled genes with a true tree that contain a specific clade (Ané et al., 2007). Thus, concordance factors are not support values and it is possible for a clade in an estimate of the species tree to exhibit strong support despite the existence of genes with histories that conflict with that clade. To obtain the input trees for BUCKy, we used MrBayes 3.1.2 (Ronquist & Huelsenbeck, 2003) on the CIPRES Science Gateway (Miller, Pfeiffer & Schwartz, 2010) for each nuclear locus or for the concatenated mitochondrial data. The appropriate model for analyses was determined by using the Akiake Information Criterion in Modeltest 3.6 (Posada & Crandall, 1998), limiting our consideration to those models implemented in MrBayes. Since the mitochondrion is a single locus, we concatenated the sequences but partitioned by mitochondrial region in the analyses. Searches were run as described above for the concatenated dataset. We also examined the BUCKy population tree, which is built by combining the set of quartets with the highest concordance factors using the Xin, Ma & Zhang (2007) algorithm. The BUCKy population tree is a consistent estimator of the species tree when discordance among gene trees reflects the multispecies coalescent (in contrast to the primary concordance tree, which is a summary of agreement among gene trees rather than an estimate of the species tree). Our second approach was to use NJ_st_ (Liu & Yu, 2011) as implemented on the Species Tree Webserver (Shaw et al., 2013). NJ_st_ uses observed internode distances on gene trees to generate a distance matrix that is then analyzed by neighbor joining, and it is also expected to be a consistent estimator of the species tree. To accommodate uncertainty in the gene tree estimates, we used the 500 bootstrap replicates from RAxML for each of the individual nuclear loci and combined mitochondrial data (see above for details) as input for the NJ_st_ analysis.

We generated datasets of varying lengths by jackknifing to explore the relationship between sequence length and the power to resolve relationships. Since the mitochondrial genome is limited in size and is thought to form a single non-recombining locus (Berlin & Ellegren, 2001; Berlin, Smith & Ellegren, 2004), obtaining larger and larger datasets will by necessity involve sampling from the nuclear genome. Thus, we only used the nuclear sequence data to generate the jackknifed datasets. This also avoided the much more rapidly evolving sites in the mitochondrial genome. We generated 100 jackknifed datasets that were 450, 550, 650, 750, 1000, 1100, 1650, 2000, 5000, 8000 and 11,000 bp (the alignment of the nuclear loci, after excluding the sites indicated above, was 12731 bp in length). This led to jackknifed datasets similar in length to the individual loci or mitochondrial regions (to allow comparison with those) as well as longer datasets to explore the affect of increasing the amount of data. For each dataset, we estimated the ML tree in PAUP^∗^, using the same approach as described above. We then examined differences between the ML tree estimated from the complete nuclear data matrix and the trees estimated from the jackknifed datasets as well as the individual partitions by calculating RF distances (Robinson & Foulds, 1981).

We also conducted Bayesian analyses Phycas (Lewis, Holder & Swofford, 2010). These analyses were focused on examining the ability of individual loci to resolve branches; Phycas implements a reversible jump Markov chain Monte Carlo (MCMC) method that assigns polytomies a prior density greater than zero (Lewis, Holder & Holsinger, 2005). We reasoned that loci with sufficient signal to resolve specific nodes would overwhelm the prior on the polytomy whereas those with more limited signal would be unable to do so and the MCMC chain would then sample trees with polytomies. From this, we obtained: (1) the number of distinct trees sampled by the MCMC chain without the polytomy prior (this is comparable to a standard Bayesian analysis that produces fully resolved trees); (2) the number of distinct trees sampled by the MCMC chain with the polytomy option; and (3) the typical resolution of trees sampled by the MCMC chain (number of resolved nodes). If polytomies are allowed in the analysis one would expect an unresolved tree to be sampled multiple times, whereas in an analysis that forbids polytomies, there may be multiple (potentially random) resolutions of that polytomy, resulting in the sampling of more distinct trees. Thus, the reduction in trees when polytomies are allowed could be viewed as an indicator of datasets with limited resolution. However, this is not expected to be the cases when a locus supports a relatively well-resolved topology. This reflects the fact that there are a substantially larger number of trees with polytomies than there are bifurcating trees (Felsenstein, 1978). When a locus has relatively high power to resolve the gene tree one might recover a single resolution for groups with relatively limited support when polytomies are forbidden but sample both the resolved and unresolved versions of the relevant branches when polytomies are allowed. This will lead to an increase in the number of trees sampled in analyses with the polytomy prior). The number of resolved nodes when polytomies are allowed can help identify cases where polytomies are prevalent (poorly resolved loci) versus rare (resolved loci). For this study we used the same models for analysis as those used for MrBayes (see above) and the same polytomy prior as Lewis, Holder & Holsinger (2005).

Finally, we explored the differences among individual loci by comparing the RAxML bootstrap results and Phycas posterior probabilities for individual loci (see above) among several clades that received high bootstrap support using either analyses of concatenated data or NJ_st_ (86–100%) but very different concordance factors (see below). Several of these nodes have been well-supported in a range of recent galliform phylogenies (e.g., Bonilla, Braun & Kimball, 2010; Cohen et al., 2012; Cox, Kimball & Braun, 2007; Crowe et al., 2006; Kaiser, van Tuinen & Ellegren, 2007; Kriegs et al., 2007; Meng et al., 2008; Nadeau, Burke & Mundy, 2007; Shen et al., 2010), including those defining the “erectile” clade (A), the “core” Phasianidae (all phasianids except the Arborophilinae, which is represented by *Rollulus* in this study) (B), Phasianidae (C), and the clade comprising Phasianidae and Odontophoridae (excluding other galliform families) (D). We included two other clades that received strong bootstrap support that have not been recovered consistently in recent studies that we also examined. One of these is the clade comprising the Argus pheasants (represented here by *Argusianus*) and the peafowl (*Pavo* and *Afropavo*) (E), and the other is a clade comprising the phasianids that are neither included in the erectile clade nor Arborophilinae (this “non-erectile clade” forms a grade in many studies) (F).

## Results and Discussion

The nuclear loci sequenced were distributed on 12 chromosomes in the chicken genome, including macro- and microchromosomes, providing both a broad sampling of the genome and sampling of loci that likely differ in patterns of molecular evolution (Axelsson et al., 2005). When the 13970 sites from the nuclear genome were combined with the 3265 sites obtained from the mitochondrial genome a total evidence data matrix of 17235 sites was obtained. The majority of sites from the nuclear genome were non-coding, with only, 208 sites from coding exons (two amplicons spanned two introns and the intervening exons were sequenced for these loci). After we excluded the hard to align regions, the microinversions, and a long autapomorphic TE insertion in FGB (a total of 1564 sites) there were 15,866 sites used in analyses (12,731 nuclear and 3135 mitochondrial). Synapomorphic TE insertions are most likely to be found on long, and uncontroversial branches (e.g., Han et al., 2011), as was true with the synapomorphic TE insertions that were included in these analyses.

The mitochondrial data had a greater proportion of parsimony informative sites (41%) than the nuclear data (36%), though there were slightly fewer variables sites in the mitochondrial data (48%) relative to the nuclear data (52%). We also observed base composition heterogeneity using the *χ*^2^ for the combined mitochondrial data (Table S3), which appeared to be driven primarily by ND2 (the only region with a *χ*^2^ *P*-value <0.05). Although the interpretation of the *χ*^2^ test of base compositional heterogeneity is complicated by the lack of independence among taxa, the very high *P*-values (all but four of the individual regions had *P* = 1.0; Table S3) strongly suggest that base composition was stable among taxa. We observed a range of values for RCV, without a clear break among the gene regions. However, we note that the top quartile (five regions) included all three mitochondrial regions and two nuclear introns (EEF2 and HMGN2). Trees based upon NJ of Euclidean distances among vectors of base composition were largely random (Supplemental Information) further suggesting that there is little potential for base composition to artificially cluster taxa. Indeed, we noted that ML trees for the five regions in the upper quartile of RCV values were not exceptionally incongruent with from the total evidence tree (see below; section entitled “*Sampling loci throughout the genome*”). Taken as a whole, these results indicate that convergence in base composition is unlikely to have had an impact on our estimates of phylogeny.

### Relationships within galliforms based upon mitochondrial data and 15 nuclear loci

**Figure 1:**
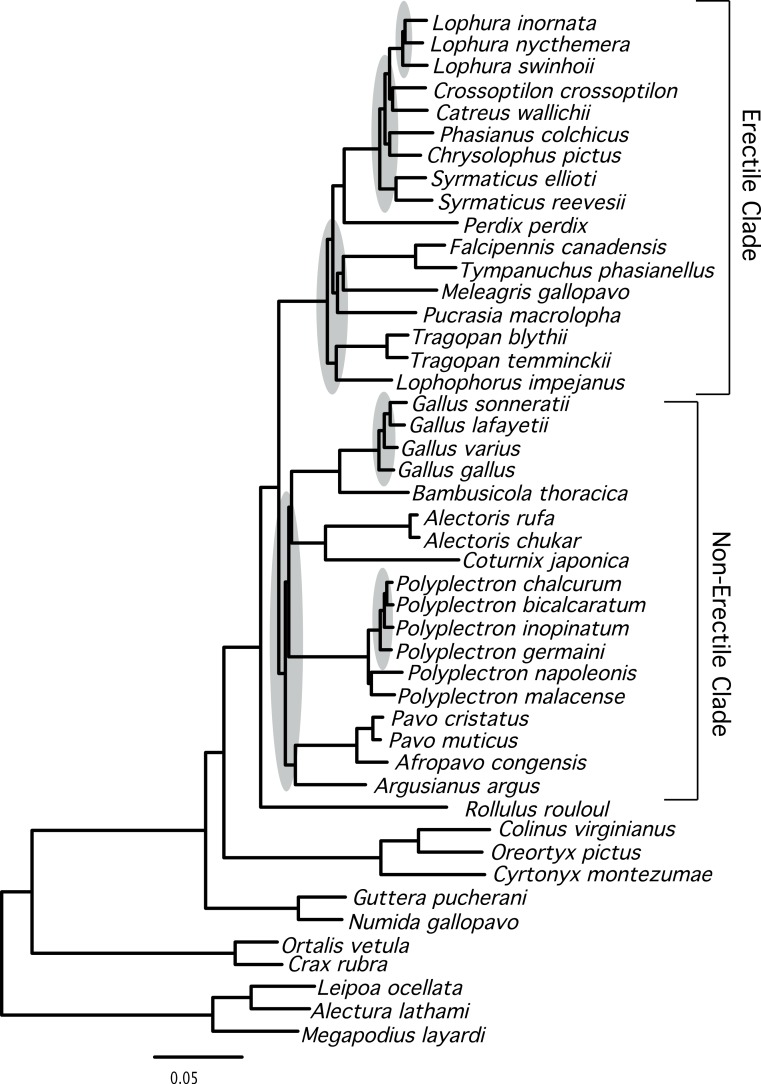
Phylogram showing branch lengths estimated using a partitioned ML tree. The tree was generated using RAxML as described in the Methods. Nodes that correspond to rapid radiations are shaded in gray. Three of the rapid radiations correspond to branches that define relationships within genera (*Lophura*, *Gallus*, and *Polyplectron*).

**Figure 2:**
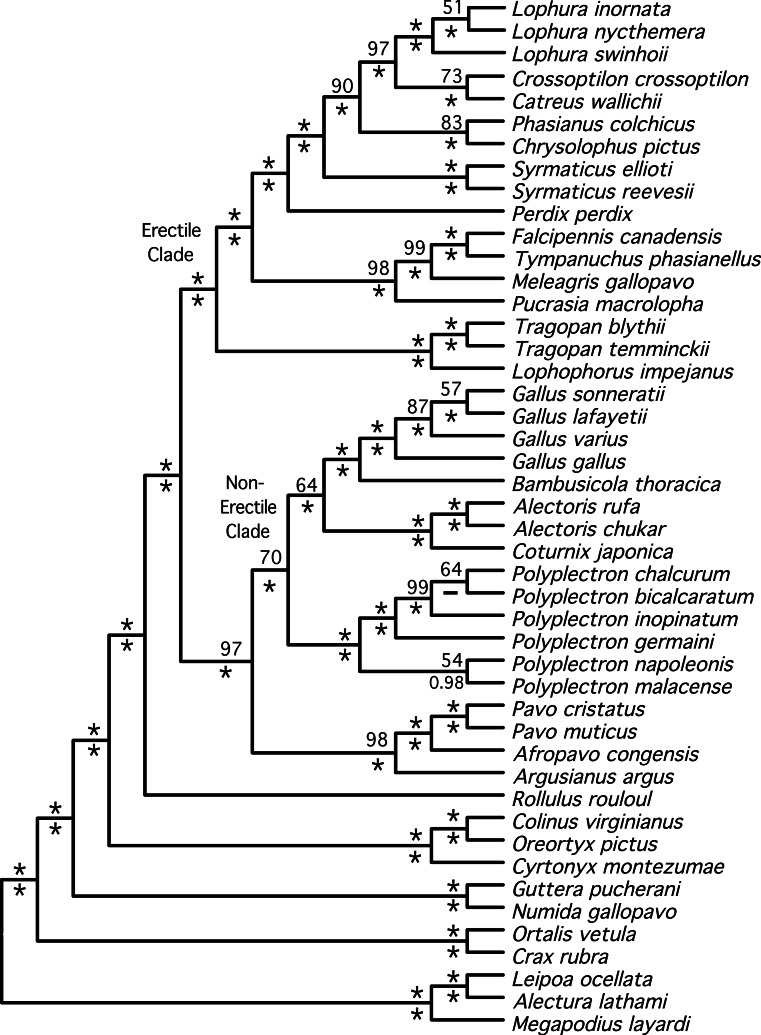
Cladogram representing the partitioned ML tree estimated from the total evidence (nuclear + mitochondrial) dataset. Values above nodes are the % bootstrap support from the partitioned ML analysis and the values below nodes are the posterior probabilities from a Bayesian analysis. ^∗^ represents either 100% bootstrap support or a posterior probability of 1.0.

The total evidence partitioned ML tree revealed a number of short branches (Fig. 1), as expected based upon prior studies (e.g., Cox, Kimball & Braun, 2007; Kimball et al., 1999; Wang et al., 2013). These branches (shaded in gray in Fig. 1) correspond to a number of clades that have either had limited support (reviewed by Wang et al., 2013) or have not been recovered in prior studies: relationships within three genera (Lophura, Gallus and Polyplectron), and three intergeneric relationships—within the erectile clade, at the base of the erectile clade (Kimball & Braun, 2008), and at the base of the non-erectile clade. Nonetheless, the total evidence partitioned ML tree (Fig. 2) had relatively high support at most nodes, particularly many of the nodes that define relationships among genera. There were some topological differences among the analyses. For example, the unpartitioned and partitioned ML analyses had three conflicting branches (RF distance = 6). However, all of these differences involved rearrangements within genera (*Gallus*, *Lophura* and *Polyplectron*). Topologically, the consensus tree from the Bayesian MCMC analysis was almost identical to the partitioned ML tree; the only difference being a rearrangement within *Polyplectron* (Fig. 2) that was not highly supported (a posterior probability of 0.94). Only one other node received a posterior probability less than 1.0, and it was also within *Polyplectron* (both of these poorly supported nodes received less than 70% support in the bootstrap analyses). Thus, outside of some conflicts among recently diverged taxa, the total evidence phylogeny was well supported and consistent among analyses.

The partitioned and unpartitioned ML trees estimated using the nuclear data were topologically identical (Supplemental Information). However, partitioning the mitochondrial data (by gene region) resulted in several differences when compared with the unpartitioned mitochondrial topology (Supplemental Information). This included two differences that were within genera (*Gallus* and *Polyplectron*) while a third difference involved relationships between *Phasianus* and *Chrysolophus*. The partitioned total evidence tree was much more similar to the tree estimated from the nuclear-only data than the mitochondrial-only data (RF distance = 2 versus 18), probably reflecting, at least in part, the fact that there was nearly 4-fold more sites in the nuclear dataset.

**Figure 3:**
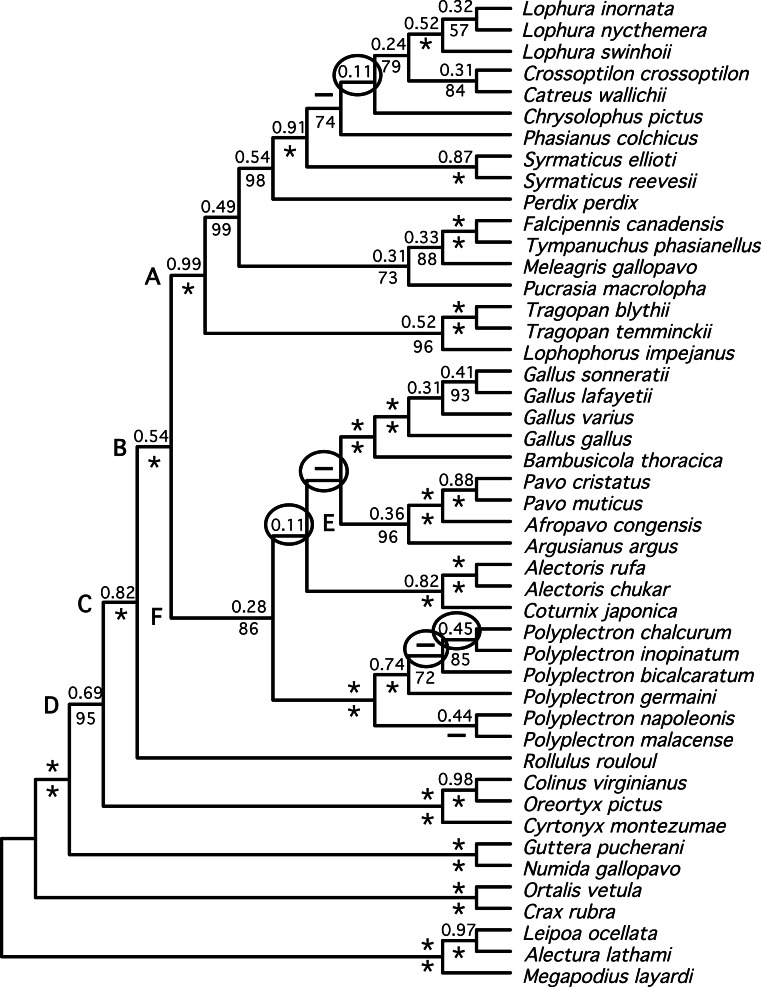
Species tree estimated from individual gene trees. Shown is the BUCKy population tree. Values above nodes are the sample-wide concordance factors, while values below the nodes are the % bootstrap support from the NJ_*st*_ analysis; ^∗^ represents either 100% bootstrap support or a concordance factor of 1.0. For the NJ_*st*_ analysis only values ≥ 50% are shown. Letters (A–F) correspond to the key nodes included in Table 1 and Table S4. Nodes that are circled represent differences between this topology and the topology obtained when partitioned analyses of concatenated data are conducted (Figs. 1 and 2). Dashed lines above the node indicate that the primary concordance tree exhibited a different topology from the BUCKy population tree and dashed lines below the node indicate that the NJ_*st*_ tree exhibited a different topology.

Both estimates of the species tree from individual gene trees (Fig. 3)—the BUCKy population tree (which should provide an unbiased estimate of the species tree if discordance among gene trees reflects the multispecies coalescent) and the NJ_st_ tree—were very similar. In fact, they differed at just a single node (within *Polyplectron*). The species trees were similar to the total evidence trees obtained by analysis of the concatenated data, being more similar to the partitioned (RF distance to the BUCKy tree = 8) than the unpartitioned (RF distance to the BUCKy tree = 14) tree. All differences to the total evidence partitioned ML topology were within the Phasianidae, although they did involve rearrangements within genera, among genera, and among higher-level clades (see circled nodes in Fig. 3). Most of the differences were at nodes with low bootstrap support in the NJ_st_ analyses, those that had low concordance factors (Fig. 3), and/or those that were absent in the primary concordance tree. These problematic relationships also corresponded to relationships that have varied among recent studies (reviewed in Wang et al., 2013) and thus represent some of the more challenging nodes within the Phasianidae that may require substantially more data from unlinked genes to resolve with confidence.

**Figure 4:**
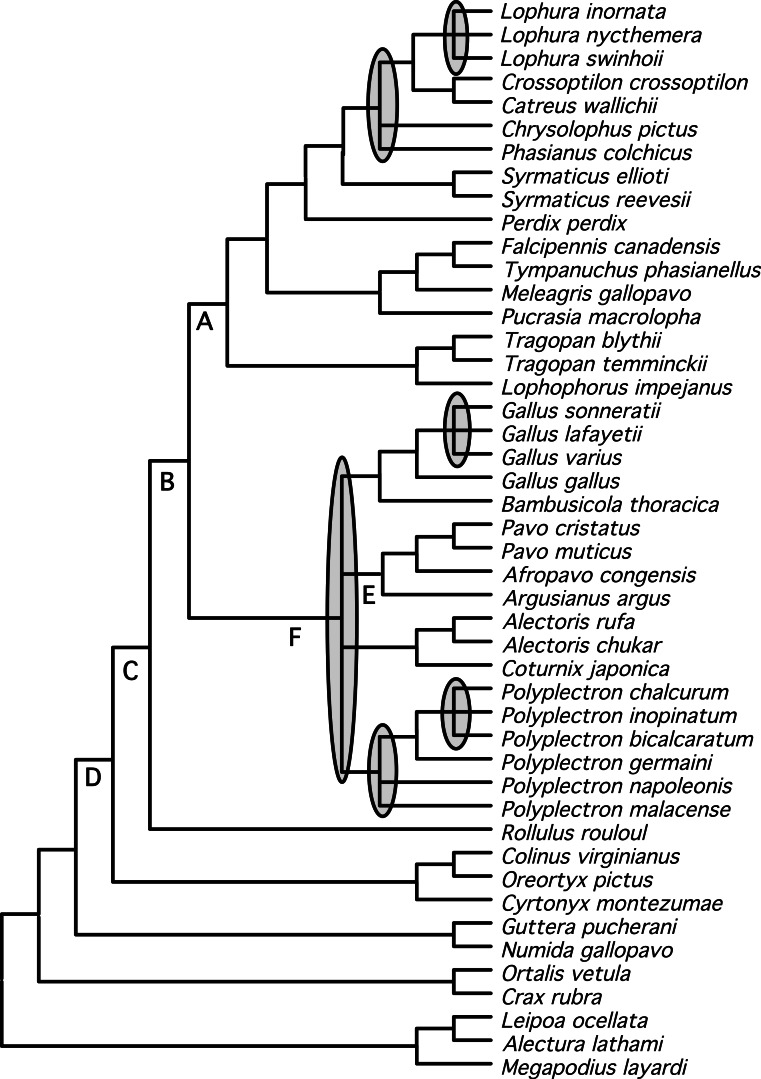
Summary tree presenting our best estimate of galliform phylogeny. Poorly supported nodes and nodes that are in conflict between Figs. 2 and 3 collapsed and shaded.

We identified a set of relationships within the Phasianidae that appeared to be robustly supported by this data (Fig. 4) by excluding those nodes that were poorly supported (those with less than 70% bootstrap support or 0.95 posterior probability) and those that were incongruent between the total evidence (Fig. 2) and species trees (Fig. 3). This set of relationships divided the “core phasianids” (the members of Phasianidae excluding Arborophilinae) into two clades: the erectile clade and the non-erectile clade. The erectile clade has been recovered in a number of studies and is often recovered with high support in analyses of individual loci (Kimball & Braun, 2008). Moreover, the use of better-fitting models increased support for this clade in analyses of a region (CYB) that does not support the clade in many analyses (Kimball et al., 2006). In contrast, the non-erectile clade was not recovered in many prior studies (reviewed by Wang et al., 2013). When it has been recovered it was either poorly supported or its recovery varied among analyses [in Wang et al. (2013), analysis of concatenated data recovered the clade whereas the estimate of the species tree obtained using NJ_st_ did not]. In this study, this major clade received >80% bootstrap support in both the species tree analysis and analyses of concatenated data. This provides strong support for the existence of a clade (the non-erectile clade) including both of the important galliform model systems [the chicken (*Gallus gallus*) and Japanese quail (*Coturnix japonica*)] and separating them from the agriculturally important turkey (*Meleagris gallopavo*), which is a member of the erectile clade.

Support for relationships within these two major clades varied. Within the erectile clade, relationships among genera were largely resolved (with the exception the positions of *Phasianus* and *Chrysolophus*), even though branch lengths at the base of this clade were short (e.g., Fig. 1). Our analyses found strong support for uniting *Meleagris* with the grouse, and those taxa with the koklass pheasant (*Pucrasia macrolopha*); the sister group of the turkey has been variable and the support for whatever relationship was found has been limited in previous studies (reviewed in Wang et al., 2013). In the non-erectile clade (Fig. 1), however, there were four well-defined clades but relationships among those four clades remained problematic. The other poorly resolved nodes (represented by polytomies in Fig. 4) were all within genera (*Lophura*, *Gallus*, and two in *Polyplectron*). In every genus where three or more species were sampled, at least some of the relationships were poorly supported (even when all, or nearly all, species within the genus were sampled such as in *Gallus* and *Polyplectron*). Rearrangements within these genera represented many of the differences among analyses of the different data partitions (total evidence, nuclear and mitochondrial) as well as the analysis of concatenated data and estimates of the species tree using methods that incorporated the multispecies coalescent. The difficulty in resolving these problematic relationships likely reflect a combination of relatively rapid radiations leading to short internodes that did not allow for sufficient substitutional variation to accumulate, and gene tree discordance due to lineage sorting. However, other processes (i.e., problems estimating the gene tree due to patterns of molecular evolution) may also contribute. The amount of additional data that will be needed to establish these relationships with confidence, if resolution is possible, is unclear.

### The impact of larger datasets

This dataset included more loci than other previously published galliform phylogenies (e.g., Bonilla, Braun & Kimball, 2010; Kimball & Braun, 2008; Nadeau, Burke & Mundy, 2007; Wang et al., 2013), but, like those other studies, there are still some nodes that lacked support. Simulations generally show that adding data initially results in a rapid approach to the true tree followed by a more gradual improvement as data continues to be added (e.g., Chojnowski, Kimball & Braun, 2008). This raises the issue of whether increasing the size of the dataset that we report here has resulted in appreciable improvements to the phylogenetic resolution within Galliformes. Specifically, are we in the relatively rapid phase of improvement or the more gradual phase?

To address this question with a direct comparison between this study and a study using less data, we conducted a partitioned ML bootstrap using the dataset assembled in this study restricting the taxa analyzed to the 44 taxa included in Kimball & Braun (2008), which included four nuclear loci and two mitochondrial regions (Supplemental Information). In general, increasing the dataset approximately 3-fold (5533 sites in Kimball & Braun (2008) compared to 15866 in this study) resulted in higher bootstrap support for a number of nodes. However, the differences were modest. There were seven nodes that increased by 5% or more in bootstrap support in the larger dataset (including two that were present but had less than 50% in Kimball & Braun, 2008), while only three nodes decreased by 5% or more. Several nodes differed, all involving relationships that remained problematic (see shaded nodes in Fig. 4).

Increasing the number of loci has also been suggested to improve analyses of species trees (e.g., Corl & Ellegren, 2013; McCormack, Huang & Knowles, 2009). Kimball & Braun (2008) only reported analyses of concatenated data, and so the results of that study could not be compared to the species tree estimated assuming the multispecies coalescent (Fig. 3). Instead, we estimated a species tree in NJ_st_ using the loci included in Kimball & Braun (2008), but with the same bootstrap input trees used in Fig. 3. This corresponded to the comparison of a tree based upon 46 taxa and 16 loci (the NJ_st_ shown in Fig. 3) to one with the same taxa but only five loci (Supplemental Information). Increasing the number of loci resulted in an increase of at least 5% bootstrap for six nodes and a decrease of at least 5% for only one node. Two nodes that received moderate to high (73% and 98%) bootstrap support in the 16-locus analysis were unresolved (e.g., received less than 50% bootstrap support) in the 5-locus species tree. As with the comparison using concatenated data, there were five nodes that were topologically different (all of these were within genera). Inclusion of a mitochondrial partition has been suggested to lead to a greater improvement relative to the addition of a nuclear locus (Corl & Ellegren, 2013), probably reflecting the greater degree of variability of the mitochondrial genome (cf. Lanier, Huang & Knowles, 2014). Therefore, we also compared the 16-locus species tree with a 15-locus tree where the mitochondrial partition was excluded. While exclusion of the mitochondrial data reduced 4 nodes by at least 5% bootstrap support, 3 other nodes increased by at least 5%. Thus, the inclusion of mitochondrial data appeared to have a modest impact upon our coalescent-based analyses. Overall, the level of improvement with the inclusion of additional data for the species tree analyses was modest and similar to that obtained in analyses of the concatenated data.

**Figure 5:**
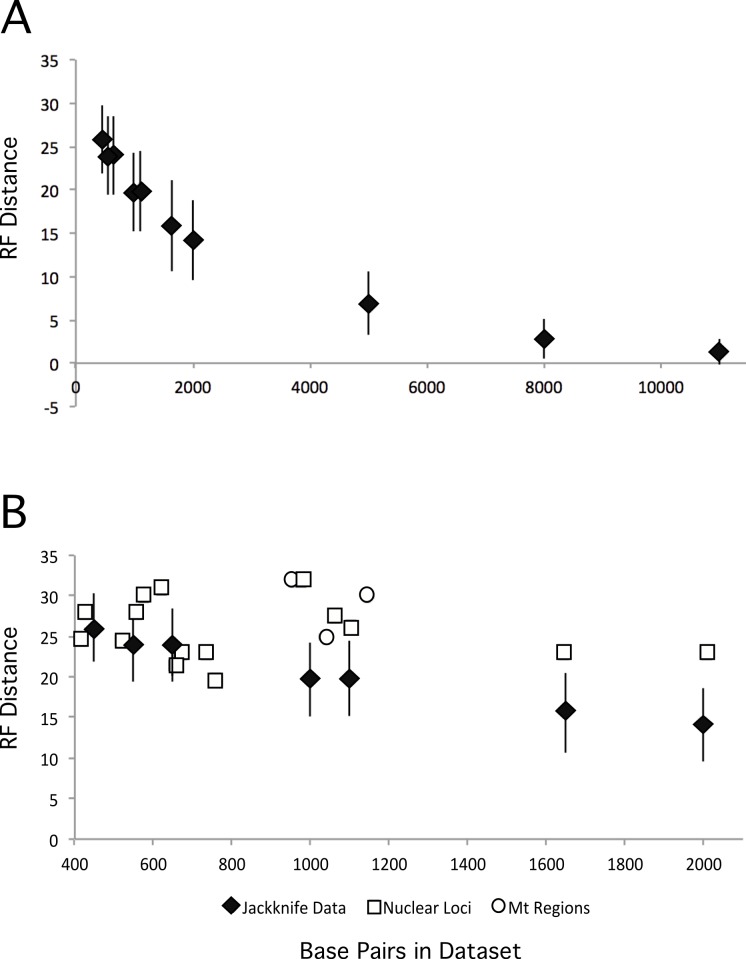
RF distances between the unpartitioned ML topology and estimates of phylogeny based upon different data matrices. Diamonds represent the average RF distance of the 100 jackknifed datasets from the ML tree and the error bars represent the standard deviation of the RF distances. (A) RF distances between jackknifed datasets and the ML tree estimated from the nuclear dataset for datasets of varying sizes. (B) RF distances between the ML trees estimated from each locus or mitochondrial region and the nuclear tree are shown.

We also explored the impact of increasing dataset size by creating jackknifed datasets of various different sizes, and comparing the ML tree from those datasets with our partitioned ML tree. As expected, increasing the numbers of base pairs in the dataset led to ML trees that were closer to the total evidence tree (Fig. 5A). However, the degree of improvement decreased as data were added and the benefits to increasing the number of loci quickly become quite limited. Thus, the relatively modest improvements that we observed are consistent with the increase in dataset size form that analyzed in Kimball & Braun (2008) to that examined here. These results suggest it may require substantially greater amounts of data (relative to the almost three-fold increase reported here) to resolve these additional nodes with confidence.

### Taxon sampling

Increased taxon sampling generally improves phylogenetic estimation (e.g., Zwickl & Hillis, 2002). This raises the question of whether additional taxa will be necessary to resolve the deep structure of galliform phylogeny. However, studies with greater taxon sampling but more limited amounts of sequence data have already been conducted. For example, Wang et al. (2013) included all species in this study along with 42 additional galliforms but only sampled six nuclear introns and two mitochondrial gene regions. These additional species were primarily in the Phasianidae, and included many genera not sampled in this study, thus subdividing many branches (particularly in the non-erectile clade). Two supermatrix studies (Crowe et al., 2006; Kimball, St Mary & Braun, 2011) had even better taxon sampling, with the latter including many more species (approximately 60% of the order and sampling all but four genera within the Phasianidae). However, in all of these three studies, which sampled well within all three of the major clades within the Phasianidae, many nodes still received low bootstrap support. Thus, increased taxon sampling may not be sufficient to resolve galliform relationships. While taxon sampling may often improve phylogenetic estimates, Sanderson & Wojciechowski (2000) noted a general decrease in bootstrap support when adding taxa, which they attributed, at least in part, to homoplasy randomly distributed among taxa. Studies based upon limited amounts of data many be especially susceptible to this effect.

### Localized biases—insights from independent evidence?

Analyzing multi-locus data matrices has been shown to result in the recovery of many strongly supported clades, even for difficult problems (e.g., Dunn et al., 2008; Hackett et al., 2008). However, there are instances where one or a few genes exhibit strong localized biases that do not reflect evolutionary history. In some cases they may reflect well-understood phenomena such as convergence in base composition (e.g., Katsu et al., 2009) while in others the biological basis for the localized bias is more obscure (e.g., Kimball et al., 2013). Regardless of their basis, these biases can affect phylogenetic signal in idiosyncratic ways. In fact, previous studies have even established that conflicting phylogenetic signals are associated with different mitochondrial regions (Cox, Kimball & Braun, 2007), even though the avian mitochondrial genome is non-recombining (Berlin & Ellegren, 2001; Berlin, Smith & Ellegren, 2004) so all regions are expected to have the same gene tree. Thus, localized biases exist in the mitochondrial genome, possibly reflecting the complex patterns of sequence evolution that make it difficult to extract phylogenetic signal from the mitochondrial genome (cf. Braun & Kimball, 2002; Powell, Barker & Lanyon, 2013). Biases can drive non-historical relationships both in analyses of concatenated data and in coalescent-based estimates of species trees (e.g., Kimball et al., 2013), making it desirable to identify localized biases, if they exist.

We have suggested that analyzing independent, multi-locus datasets (i.e., those with no overlapping loci between datasets) can help identify nodes that may be present due to localized biases (Kimball et al., 2013). If no conflict is identified, analysis of independent datasets can lead to increased confidence in relationships and greater justification for combining datasets across different studies (e.g., Smith, Braun & Kimball, 2013; Wang, Braun & Kimball, 2012). To conduct such an independent evidence analysis for Galliformes we compared the results from Kimball & Braun (2008) with an ML analysis of the 11 nuclear loci and 1 mitochondrial region in this study that were not included in the Kimball & Braun (2008) dataset. Although the mitochondrial regions form a single locus, analyses of distinct mitochondrial regions can result in different topologies (e.g., Cox, Kimball & Braun, 2007), presumably due to distinct patterns of molecular evolution in each region. Therefore, we included the mitochondrial 12S rRNA in the independent evidence data matrix along with the 11 nuclear loci. This data matrix was limited to the 44 taxa that were included in Kimball & Braun (2008), and a comparison of the ML analysis of this independent data matrix revealed several differences from the Kimball & Braun (2008) tree (Supplemental Information). However, all differences involved relationships supported at less than 70% (often less than 50%) bootstrap support, suggesting there is little or no conflicting signal between the two datasets. The topology and levels of support were also very similar when the 44-taxon tree (which allowed a direct comparison with Kimball & Braun, 2008) and the 46-taxon tree were compared. Thus, this analysis did not reveal any evidence for localized biases that might affect our conclusions and indicate that including all loci is justified.

### Sampling loci throughout the genome

Expanded sampling of loci has the potential to reduce the impact of discordance among gene trees by providing a better sample of the topologies that resulted from the multispecies coalescent during evolutionary radiations. To determine whether sampling more loci (versus sampling the same number of total sites but from fewer loci) is advantageous we compared the performance of individual loci (and mitochondrial regions) against jackknifed datasets of comparable size (Fig. 5B). The jackknifed datasets represent sites sampled from all loci, and thus include a mixture of sites that come from loci that are evolving at different rates, exhibit different patterns of evolution, and different evolutionary histories (i.e., distinct gene trees). Although analyses of six loci yielded trees that were at least as similar to the total evidence tree as the average jackknifed dataset (GAPDH, OVM, HSP90B1, CHRNG, CLTC, and HMGN2), the other loci (and all three of the mitochondrial regions) yielded gene trees that were more divergent from the total evidence tree than the jackknifed datasets of similar sizes. Not only did the majority of loci perform worse with respect to recovering the total evidence tree than average samples of sites from the data matrix, they typically performed substantially worse. Thus, on average, for a given number of base pairs, sampling sites from many loci around the genome appears to perform better than sampling from one or a few loci.

There are several reasons why sampling multiple loci is likely to be advantageous. It has long been recognized that patterns of molecular evolution may make different loci ideal for the resolution of nodes at different depths in the tree (Graybeal, 1994). Unlinked genes are also expected to have somewhat distinct evolutionary histories (Oliver, 2013), so inclusion of many different loci provides more information about these differences among gene trees. Finally, broader sampling of loci should reduce the impact of localized biases, if they are present. The relative contributions of these phenomena to the observed incongruence among estimates of gene trees can be difficult to establish, though an examination of the performance of individual loci can illustrate the differences among loci. For example, when several key clades that received high bootstrap support (97–100%) were examined we found substantial variation in the concordance factors (nodes labeled A–F, Fig. 3). Different nuclear loci and mitochondrial gene regions show substantial variation in whether these key clades are found, and if found, the levels of bootstrap support (Table 1). Thus, it appears that the specific clades supported by a locus are quite variable—some loci supported one clade whereas other loci provided signal supporting other clades. Consistent with our previous results (Fig. 5A), the two longest loci (FGB and SERPINB14; Table 2) supported the greatest number of clades with at least 50% bootstrap support, while a relatively short locus (OVM) supports none of these clades with at least 50% bootstrap support. However, we note that the RF distance from the total evidence tree (Fig. 2) was not substantially lower for the longest loci than it was for the shorter loci (Fig. 5A). This finding is consistent with the hypothesis that at least some of the differences between individual gene trees and the species tree reflect discordance due to the coalescent rather than the lack of power due to their short length. Although at least some genuine differences among gene trees due to incomplete lineage sorting during speciation events is expected, even those deep in the tree (Oliver, 2013; Patel, Kimball & Braun, 2013), it is far from clear how much observed incongruence among gene trees reflects discordance rather than estimation error for galliform phylogeny (just as it is for most empirical studies in phylogenetics).

**Table 1:** Support for key nodes in galliform phylogeny. Letters refer to the clades labeled in Fig. 4. Bootstrap support ≥ 50% is indicated in bold; concordance factors are not bolded since they provide information different from support (see text). Dashes indicate that the clade of interest was not recovered.

	A	B	C	D	E	F
Bootstrap, Concatenation (Fig. 1)	100	100	100	100	98	97
Concordance factor (Fig. 2)	0.99	0.54	0.82	0.69	0.36	0.28
Bootstrap, NJst (Fig. 2)	100	100	100	95	96	86
ALDOB	**92**	25	**62**	**75**	–	–
CALB1	**93**	–	35	–	–	33
CHRNG	**97**	–	36	–	49	–
CLTC	**97**	**93**	**96**	**78**	–	–
CLTCL1	**76**	–	**92**	**93**	–	–
CRYAA	**100**	**90**	**99**	**95**	34	–
EEF2	**95**	–	–	**100**	–	–
FGB	**100**	**97**	**100**	**100**	**72**	38
GAPDH	**96**	–	**77**	**83**	38	–
HMGN2	**100**	36	**86**	–	49	45
HSP90B1	**100**	**91**	**76**	**94**	–	–
OVM	28	–	–	47	–	–
PCBD1	**89**	**73**	**74**	**57**	–	17
RHO	**100**	**50**	**98**	–	–	**56**
SERPIN	**100**	48	**100**	**63**	**76**	**66**
ND2	**97**	**50**	**84**	39	21	17
CYB	**60**	**64**	**73**	42	–	–
12S	**90**	–	36	**51**	**60**	–

**Table 2:** Power of individual loci and mitochondrial regions to resolve galliform phylogeny based upon analyses using Phycas. The length, number of variable sites, and number of parsimony informative sites for each region is presented along with the results of analyses in Phycas. The Phycas results are the percentage of unique trees sampled when the MCMC chain can (+) or can not (−) sample trees with polytomies and the proportion of resolved nodes when polytomies can be sampled. The mean and range are reported for the latter; a value of 1.0 would indicate a fully resolved tree. Support for specific clades is reported in Table S4.

	Length	Variable	Informative	% Unique trees sampled		Resolution
	(bp)	sites	sites	(Polytomy ±)		
Locus				−	+	
ALDOB	555	329	216	100.0	99.8	0.65 (0.63–0.67)
CALB1	623	260	159	100.0	100.0	0.63 (0.6–0.65)
CHRNG	673	262	167	99.4	96.3	0.72 (0.7–0.74)
CLTC	735	396	287	17.0	32.3	0.86 (0.84–0.88)
CLTCL1	427	260	173	100.0	94.8	0.7 (0.67–0.72)
CRYAA	1061	582	396	96.5	69.6	0.74 (0.74–0.77)
EEF2	975	329	261	94.7	94.0	0.77 (0.74–0.79)
FGB	1645	952	694	29.3	49.4	0.79 (0.77–0.81)
GAPDH	417	238	172	100.0	99.1	0.72 (0.7–0.74)
HMGN2	758	501	399	97.2	96.9	0.74 (0.72–0.77)
HSP90B1	656	427	324	94.7	95.6	0.77 (0.74–0.79)
OVM	519	253	168	100.0	99.5	0.67 (0.65–0.7)
PCBD1	575	357	236	99.9	98.6	0.74 (0.72–0.77)
RHO	1105	647	494	86.8	83.6	0.74(0.72–0.77)
SERPIN	2007	729	439	45.9	39.3	0.74 (0.72–0.77)
ND2	1041	604	523	95.0	99.1	0.79 (0.77–0.81)
CYB	1143	543	473	94.8	94.2	0.81 (0.79–0.84)
12S	951	345	282	100.0	99.9	0.72 (0.67–0.74)

Emphasizing the role of stochastic error in gene tree estimation, we note that simulations using parameters appropriate for birds recovered only 50%–75% of the very short nodes in avian phylogeny when introns similar in length to those used here were used (Chojnowski, Kimball & Braun, 2008). Although individual gene trees are expected to be bifurcating (Slowinski, 2001), it is possible that the branches in a gene tree may be short enough that the probability of even a single synapomorphic substitution along those branches is very low (cf. Braun & Kimball, 2001). To determine whether some of the trees associated with the nuclear loci and mitochondrial gene regions that we used supported trees that were effectively polytomies, we compared the ability of Bayesian analyses in which polytomies are allowed to analyses producing fully bifurcating trees. Large numbers of distinct trees were sampled regardless of whether or not polytomies were allowed in the analysis, but in most cases fewer trees were sampled when polytomies were allowed (Table 2), as would be expected if individual gene trees were poorly resolved. There were two exceptions (CLTC and FGB) where the analyses allowing polytomies sampled more trees, suggesting that those sequences (both of which were relatively long; Table 2) have greater power to resolve the gene tree associated with those loci. However, none of the trees sampled were fully resolved, even for the regions with the most power to resolve relationships. Placing some prior density on polytomies also reduced the support most of the six focal nodes that we examined (Table S4), further emphasizing the limited power of individual gene regions.

Despite the limited power of the individual regions, combining these loci resulted in a well-supported phylogeny for many clades (Figs. 2 and 3). Not surprisingly, the number of loci or regions that support a specific clade does appear related to the concordance factor, which is an estimate of the proportion of sampled genes that support a clade (and, since the gene regions used here were chosen randomly, it is likely to reflect the proportion of the genome that supports a clade). However, there were clades with low concordance factors that received high support in both the analysis of concatenated data (Fig. 2) and, more significantly, the species tree analysis (Fig. 3), emphasizing the distinction between concordance factors and support values. Finally, we note that although FGB exhibits localized biases that appear to provide non-historical signal for some avian relationships (e.g., Kimball et al., 2013; Mayr, 2011) it appears to perform well in galliforms. Although the ability to resolve relationships within genera remains unclear, the increased support relative to prior studies for deep nodes in galliform phylogeny suggest that adding additional loci have the potential to resolve the backbone of this tree, even without much additional taxon sampling.

## Conclusions

A consensus regarding many relationships within the Phasianidae now appears to be emerging, despite the fact that some challenges remain before we obtain a phylogenetic tree for this group that is both well resolved and strongly supported. While taxon sampling may help in some cases (e.g., Wang et al., 2013), we have shown here that adding loci is also extremely important. Regarding nodes that have been problematic in previous studies, we found strong evidence for a second major clade within the core phasianids (the non-erectile clade). We also found strong support for uniting the grouse and turkey (*Meleagris*) within the erectile clade and for placing the koklass (*Pucrasia*) sister to that grouse-turkey clade. As expected, however, the degree of improvement with increasing dataset size was modest, reflecting the difficulty of the relationships that remain unresolved. Indeed, some relationships remain problematic even with the larger dataset used in this study. Many of these problematic relationships were within genera, though a few poorly supported relationships among the higher-level clades still remain (Fig. 4). Whether the remaining unresolved nodes represent hard polytomies, that cannot be resolved, or soft polytomies that might be resolved with even larger datasets remains to be determined. Given the modest improvement evident in this study relative to Kimball & Braun (2008), it will likely require a much larger dataset, perhaps one with an order of magnitude increase in the number of variable sites, before it becomes clear whether the remaining unresolved nodes represent hard polytomies or soft polytomies that can eventually be resolved.

## Supplemental Information

10.7717/peerj.361/supp-1 Table S1 Species examined

10.7717/peerj.361/supp-2 Table S2 Locus information

10.7717/peerj.361/supp-3 Table S3 Base composition of variable sites Results from the *χ*^2^ test of deviation from base composition homogeneity, and RCV (top quartile is indicated by * since there is no associated test statistic).

10.7717/peerj.361/supp-4 Table S4 Support for key nodes in galliform phylogeny based upon analyses in Phycas Letters refer to the clades labeled in Fig. 4. Values are posterior probabilities estimated when polytomies cannot be sampled (to the left) or can be sampled (to the right). Posterior probabilities ≥ 0.5 is indicated in bold and dashes indicate that the clade of interest was not sampled. In all cases the value reported is the proportion of times the clade of interest was sampled; clades with a posterior probability > = 0.5 may not be present in the extended majority rule consensus tree.

10.7717/peerj.361/supp-5 Supplemental Information 5 Details of treefiles

10.7717/peerj.361/supp-6 Supplemental Information 6 Treefiles

## Additional Information and Declarations

### DNA Deposition

The following information was supplied regarding the deposition of DNA sequences: Genbank KC749476–KC749685, KC749760–KC749857, KC749907–KC749953.
