# Supplementary material for: Does more sequence data improve estimates of galliform phylogeny? Analyses of a rapid radiation using a complete data matrix
Source: PeerJ. 2014 Apr 22;2:e361. doi: 10.7717/peerj.361 (PMC4006227; doi:10.7717/peerj.361)
Supplement: Table S1 [file peerj-02-361-s001.docx]

**Supplementary Table 1.** Species examined.

| Family | Species | Common name | Sources |
| --- | --- | --- | --- |
| Megapodiidae | *Alectura lathami* | Australian Brush-Turkey | UWBM 55907 |
|  | *Leiopoa ocellata* | Malleefowl | UWBM 62959 |
|  | *Megapodius layardi* | Vanuatu Scrubfowl | S. Birks |
| Cracidae | *Crax rubra* | Great Curassow | LSUMNS B-14200 |
|  | *Ortalis vetula* | Plain Chachalaca | RTK476 |
| Numididae | *Guttera pucherani* | Crested Guineafowl | LSUMNS B-20771 |
|  | *Numida meleagris* | Helmeted Guineafowl | RTK466 |
| Odontophoridae | *Colinus virginianus* | Northern Bobwhite | RTK412 |
|  | *Cyrtonyx montezumae* | Montezuma Quail | RTK420 |
|  | *Oreortyx pictus* | Mountain Quail | RTK474 |
| Phasianidae | *Afropavo congensis* | Congo Peacock | RTK382 |
|  | *Alectoris chukar* | Chukar | RTK383 |
|  | *Alectoris rufa* | Red-legged Partridge | RTK384 |
|  | *Argusianus argus* | Great Argus | LSUMNS B-13314 |
|  | *Bambusicola thoracica* | Chinese Bamboo-Partridge | RTK389 |
|  | *Catreus wallichi* | Cheer Pheasant | RTK408 |
|  | *Chrysolophus pictus* | Golden Pheasant | RTK410 |
|  | *Coturnix coturnix* | Common Quail | RTK414 |
|  | *Crossoptilon crossoptilon* | White Eared-Pheasant | RTK419 |
|  | *Dendragapus Canadensis* | Spruce Grouse | RTK422 |
|  | *Gallus gallus* | Red Junglefowl | RTK430 |
|  | *Gallus lafayetii* | Ceylon Junglefowl | RTK432 |
|  | *Gallus sonneratii* | Gray Junglefowl | RTK433 |
|  | *Gallus varius* | Green Junglefowl | RTK438 |
|  | *Lophophorus impejanus* | Himalayan Monal | RTK444 |
|  | *Lophura inornata* | Salvadori's Pheasant | RTK450 |
|  | *Lophura nycthemera* | Silver Pheasant | RTK452 |
|  | *Lophura swinhoii* | Swinhoe's Pheasant | RTK453 |
|  | *Meleagris gallopavo* | Wild Turkey | RTK462 |
|  | *Pavo cristatus* | Indian Peafowl | RTK477 |
|  | *Pavo muticus* | Green Peafowl | RTK478 |
|  | *Perdix perdix* | Gray Partridge | RTK480 |
|  | *Phasianus colchicus* | Ring-necked Pheasant | RTK481 |
|  | *Polyplectron bicalcaratum* | Gray Peacock-Pheasant | RTK483 |
|  | *Polyplectron chalcurum* | Bronze-tailed Peacock-Pheasant | RTK484 |
|  | *Polyplectron napoleonis* | Palawan Peacock-Pheasant | RTK485 |
|  | *Polyplectron germaini* | Germain's Peacock-Pheasant | RTK486 |
|  | *Polyplectron inopinatum* | Mountain Peacock-Pheasant | RTK487 |
|  | *Polyplectron malacense* | Malayan Peacock-Pheasant | RTK488 |
|  | *Pucrasia macrolopha* | Koklass Pheasant | RTK490 |
|  | *Rollulus rouloul* | Crested Partridge | LSUMNS B-24971 |
|  | *Syrmaticus ellioti* | Elliot's Pheasant | RTK493 |
|  | *Syrmaticus reevesii* | Reeves's Pheasant | RTK496 |
|  | *Tragopan blythii* | Blyth's Tragopan | RTK502 |
|  | *Tragopan temminckii* | Temminck's Tragopan | RTK527 |
|  | *Tympanuchus phasianellus* | Sharp-tailed Grouse | RTK533 |
