# Supplementary material for: Does more sequence data improve estimates of galliform phylogeny? Analyses of a rapid radiation using a complete data matrix
Source: PeerJ. 2014 Apr 22;2:e361. doi: 10.7717/peerj.361 (PMC4006227; doi:10.7717/peerj.361)
Supplement: Table S2 [file peerj-02-361-s002.docx]

| Locus | Chrom. | Intron(s) | Analyzed Length^a^ | Primer sequences or sequence source |
| --- | --- | --- | --- | --- |
| ALDOB | Z | 6 | 555 | Cox et al. 2007 (as AldB) |
| CALB1 | 2 | 9, 10 | 623 | Cox et al. 2007 (as Cal) |
| CHRNG | 9 | F | 673 | CAACCTGCTGCTGACAGTGGAGGANGGNCA  ATCTTCCGTGCTGGNCGGTGCRTGATGG |
| CLTC | 19 | 7 | 735 | Kimball et al. 2009 |
| CLTCL1 | 15 | 7 | 427 | Kimball et al. 2009 |
| CRYAA | 1 | 2 | 1061 | CATGCTGGATGTAAAACACTTYTCCC  GCCWGAGAAGGTCAGCATNCCRTC |
| EEF2 | 28 | 5, 6 | 975 | Kimball et al. 2009 |
| FGB | 4 | 5, 7 | 1645 | Kimball et al. 2009 |
| GAPDH | 1 | 11 | 417 | Cox et al. 2007 ^b^ (as G3PDH) |
| HMGN2 | 23 | 2, 3 | 758 | Cox et al. 2007 (as HMG) |
| HSP90B1 | 1 | 7 | 656 | GGAGCAAACAGATGATGAYGARGC  GGTTTTATGTCATTCATNAGRTCCC |
| OVM | 13 | G | 519 | Cox et al. 2007 (as OVOG) |
| PCBD1 | 6 | 3 | 575 | Cox et al. 2007 (as DCOH) |
| RHO | 12 | 1 | 1105 | Cox et al. 2007 (as Rhod) |
| SERPINB14 | 2 | C, D | 2007 | GTTCGCTTTGATAAACTTCCAG  GGTGATTTGGTTGAGNATGTC  GAGACATCCTCAACAAATCAC  CTCTGGCTTGATCTGCAGCTG |
| CYB | Mt |  | 1143 | Cox et al. 2007 ^b^ |
| ND2 | Mt |  | 1041 | Cox et al. 2007 ^b^ |
| 12S  (MT-RNR1) | Mt |  | 951 | Cox et al. 2007 ^b^ |

^a^ Values are sites after excluding hard to align sites, microinversions, or the insertion if FGB.

^b^ Primers we used were identical to those in this publication; the primers, or slightly modified versions of them, were originally taken from: Friesen et al. 1997, Prychitko and Moore 1997, Kimball et al. 1999, Sorenson et al. 1999, and Armstrong et al. 2001.
