# Supplementary material for: Does more sequence data improve estimates of galliform phylogeny? Analyses of a rapid radiation using a complete data matrix
Source: PeerJ. 2014 Apr 22;2:e361. doi: 10.7717/peerj.361 (PMC4006227; doi:10.7717/peerj.361)
Supplement: Table S3 — Results from the χ2 test of deviation from base composition homogeneity, and RCV (top quartile is indicated by * since there is no associated test statistic). [file peerj-02-361-s003.docx]

| Dataset | π_A_ | π_C_ | π_G_ | π_T_ | χ^2^ *P*-value | RCV |
| --- | --- | --- | --- | --- | --- | --- |
| All nuclear | 0.265 | 0.221 | 0.240 | 0.274 | 1.0 | 0.0116 |
| All mitochondrial | 0.333 | 0.405 | 0.077 | 0.185 | < 0.001 | 0.0470 |
| ALDOB | 0.280 | 0.253 | 0.186 | 0.281 | 1.0 | 0.0444 |
| CALB1 | 0.278 | 0.194 | 0.175 | 0.353 | 1.0 | 0.0369 |
| CHRNG | 0.211 | 0.325 | 0.271 | 0.193 | 1.0 | 0.0411 |
| CLTC | 0.307 | 0.207 | 0.224 | 0.262 | 1.0 | 0.0415 |
| CLTCL1 | 0.268 | 0.259 | 0.186 | 0.287 | 1.0 | 0.0394 |
| CRYAA | 0.265 | 0.222 | 0.252 | 0.262 | 1.0 | 0.0358 |
| EEF2 | 0.206 | 0.230 | 0.239 | 0.325 | 0.801 | 0.0677* |
| FGB | 0.291 | 0.183 | 0.222 | 0.305 | 1.0 | 0.027 |
| GAPDH | 0.274 | 0.217 | 0.289 | 0.22 | 1.0 | 0.0489 |
| HMGN2 | 0.248 | 0.189 | 0.269 | 0.294 | 0.69 | 0.0576* |
| HSP90B1 | 0.303 | 0.145 | 0.262 | 0.29 | 1.0 | 0.045 |
| OVM | 0.247 | 0.246 | 0.257 | 0.249 | 1.0 | 0.0419 |
| PCBD1 | 0.226 | 0.257 | 0.284 | 0.232 | 1.0 | 0.0422 |
| RHO | 0.211 | 0.264 | 0.276 | 0.248 | 1.0 | 0.037 |
| SERPIN | 0.302 | 0.219 | 0.213 | 0.265 | 1.0 | 0.0275 |
| ND2 | 0.366 | 0.38 | 0.0604 | 0.184 | 0.003 | 0.0574* |
| CYB | 0.306 | 0.471 | 0.0589 | 0.164 | 0.24 | 0.0494* |
| 12S | 0.317 | 0.325 | 0.138 | 0.221 | 1.0 | 0.0566* |
