# Supplementary material for: Does more sequence data improve estimates of galliform phylogeny? Analyses of a rapid radiation using a complete data matrix
Source: PeerJ. 2014 Apr 22;2:e361. doi: 10.7717/peerj.361 (PMC4006227; doi:10.7717/peerj.361)
Supplement: Table S4 — Letters refer to the clades labeled in Fig. 4. Values are posterior probabilities estimated when polytomies cannot be sampled (to the left) or can be sampled (to the right). Posterior probabilities ≥ 0.5 is indicated in bold and dashes indicate that the clade of interest was not sampled. In all cases the value reported is the proportion of times the clade of interest was sampled; clades with a posterior probability > = 0.5 may not be present in the extended majority rule consensus tree. [file peerj-02-361-s004.docx]

|  | Posterior probabilities for the clade of interest  (-/+ polytomy) | | | | | |
| --- | --- | --- | --- | --- | --- | --- |
| Locus | A | B | C | D | E | F |
| ALDOB | **1/1** | 0.28/0.05 | **0.90/0.84** | **0.88/0.61** | 0.01/0 | 0.06/0.01 |
| CALB1 | **1/1** | 0.23/0.07 | **0.51**/0.21 | 0.04/0.02 | 0.11/0.01 | 0.24/0.06 |
| CHRNG | **1/1** | ---/--- | **0.82/0.66** | 0.15/0.03 | **0.63**/0.17 | ---/--- |
| CLTC | **1/1** | **1/1** | **1/1** | **0.60**/0.11 | ---/--- | ---/--- |
| CLTCL1 | **1/0.99** | 0.13/0.04 | **1/1** | **0.93**/0.49 | 0.25/0.11 | 0/0 |
| CRYAA | **1/1** | **1/1** | **1/1** | **0.98/0.64** | 0.32/0.03 | 0.35/0.06 |
| EEF2 | **1/1** | 0.20/0.05 | 0.03/0.02 | **1/1** | 0.19/0.09 | ---/--- |
| FGB | **1/1** | **1/1** | **1/1** | **1/0.99** | **0.89**/0.27 | 0.39/0.08 |
| GAPDH | **1/1** | 0.13/0.03 | **1/1** | **0.86**/0.36 | **0.50**/0.36 | 0.01/0.01 |
| HMGN2 | **1/1** | 0.39/0.46 | **0.98/0.90** | 0.21/0.03 | **0.84/0.71** | **0.95/0.71** |
| HSP90B1 | **1/1** | **1/1** | **0.72**/0.19 | **1/1** | 0.06/0.05 | 0.05/0.03 |
| OVM | **0.75/0.8** | 0/0 | 0.03/0.01 | **0.89/0.64** | 0.06/0.01 | 0/0 |
| PCBD1 | **1/1** | **1/0.98** | **0.99/0.96** | **0.84/0.66** | 0.02/0.01 | 0.32/0.09 |
| RHO | **1/1** | **0.75**/0.28 | **1/1** | **0.50**/0.32 | 0/0 | **0.99/0.99** |
| SERPIN | **1/1** | **0.54**/0.04 | **1/1** | **0.68**/0.17 | **1/1** | **1/1** |
| ND2 | **1/1** | **0.99/0.89** | **1/1** | 0.29/0.18 | 0.02/0.02 | 0/0.01 |
| CYB | **1/1** | **0.87/0.75** | **1/1** | 0.2/0.1 | 0.19/0.04 | 0.27/0.09 |
| 12S | **1/1** | **0.62/0.67** | **0.61**/0.41 | **0.51**/0.23 | **0.78**/0.16 | 0/--- |
